# Supplementary material for: Predicted impact of the viral mutational landscape on the cytotoxic response against SARS-CoV-2
Source: PLoS Comput Biol. 2022 Feb 10;18(2):e1009726. doi: 10.1371/journal.pcbi.1009726 (PMC8830725; doi:10.1371/journal.pcbi.1009726)
Supplement: S1 Fig — (DOCX) [file pcbi.1009726.s001.docx]

**S1 Fig. Correspondence between netMHCpan 4.1 EL and BA scores**. Three random SARS-CoV-2 HLA class I nonamers predicted by netMHCpan 4.1 EL for each 0.005 score interval between 0 and 1 were selected. Binding of the same nonamer dataset was re-calculated for the same allele using netMHCpan 4.1 BA.
